# Oral contraceptive use and ovarian cancer risk among carriers of BRCA1 or BRCA2 mutations

**DOI:** 10.1038/sj.bjc.6602239

**Published:** 2004-11-16

**Authors:** A S Whittemore, R R Balise, P D P Pharoah, R A DiCioccio, Kathleen Cuningham Foundation Consortium for Research into Familial Breast Cancer (kConFab), I Oakley-Girvan, S J Ramus, M Daly, M B Usinowicz, K Garlinghouse-Jones, B A J Ponder, S Buys, R Senie, I Andrulis, E John, J L Hopper, M S Piver

**Affiliations:** 1 Department of Health Research and Policy, Stanford University School of Medicine, HRP Redwood Building, Stanford, CA 94305-5405, USA; 2 Strangeway's Research Laboratories, Wort's Causeway, Cambridge CB1 8RN, UK; 3 Department of Cancer Genetics and the Gilda Radner Familial Ovarian Cancer Registry, Roswell Park Cancer Institute, Elm and Carlton Streets, Buffalo, NY 14263, USA; 4 Peter MacCallum Cancer Centre, St Andrews Place, East Melbourne, Victoria 3002, Australia; 5 Northern California Cancer Center, 32960 Alvarado-Niles Road, Suite 600, Union City, CA 94587, USA; 6 Translational Research Laboratories, Department of Gynaecological Oncology, University College London, Windeyer Building, 46 Cleveland Street, London W1T 4JF, UK; 7 Cancer Prevention and Control Program, Fox Chase Cancer Center, 7701 Burholme Avenue, Philadelphia, PA 19111, USA; 8 CRC Department of Oncology, University of Cambridge, Hutchison/MRC Research Centre, Hills Road, Cambridge CB2 2XZ, UK; 9 Huntsman Cancer Institute, 2000 Circle of Hope, Salt Lake City, UT 84112, USA; 10 Mailman School of Public Health, Columbia University, 622 W 168th Street, Room PH-18-201, New York, NY 10032, USA; 11 Ontario Cancer Genetics Network, Cancer Care Ontario, 620 University Avenue, Toronto, Ontario M5G 2L7, Canada; 12 Centre for Genetic Epidemiology, University of Melbourne, Level 2,723 Swanston Street, Carlton, Victoria 3053, Australia

**Keywords:** BRCA1, BRCA2, oral contraceptives, ovarian cancer

## Abstract

Women with mutations of the genes BRCA1 or BRCA2 are at increased risk of ovarian cancer. Oral contraceptives protect against ovarian cancer in general, but it is not known whether they protect against the disease in carriers of these mutations. We obtained self-reported lifetime histories of oral contraceptive use from 451 women who carried mutations of BRCA1 or BRCA2. We used conditional logistic regression to estimate the odds ratios associated with oral contraceptive use, comparing the histories of 147 women with ovarian cancer (cases) to those of 304 women without ovarian cancer (controls) who were matched to cases on year of birth, country of residence and gene (BRCA1 *vs* BRCA2). Reference ages for controls had to exceed the ages at diagnosis of their matched cases. After adjusting for parity, the odds-ratio for ovarian cancer associated with use of oral contraceptives for at least 1 year was 0.85 (95 percent confidence interval, 0.53–1.36). The risk decreased by 5% (1–9%) with each year of use (*P* for trend=0.01). Use for 6 or more years was associated with an odds-ratio of 0.62 (0.35–1.09). These data support the hypothesis that long-term oral contraceptive use reduces the risk of ovarian cancer among women who carry mutations of BRCA1 or BRCA2.

Women who carry deleterious mutations of the genes BRCA1 or BRCA2 are at increased risk of developing ovarian cancer (Struewing *et al*, 1997; Ford *et al*, 1998, Antoniou *et al*, 2003). Oral contraceptive use is associated with reduced ovarian cancer risk in the general population (Whittemore *et al*, 1992; La Vecchia and Franceschi, 1999). It is important to know if a similar association holds for mutation carriers. Previous investigations have addressed this question with conflicting results (Narod *et al*, 1998, 2001a; Modan *et al*, 2001; McGuire *et al*, 2004). Resolution of this issue is important because oral contraceptive use at early ages may increase the risk of breast cancer in mutation carriers (Ursin *et al*, 1997; Narod *et al*, 2002).

We report the results of an analysis of ovarian cancer risk in relation to oral contraceptive use among 451 carriers of BRCA1 or BRCA2 mutations, comprising 147 women with ovarian cancer (cases) and 304 women without ovarian cancer (controls) who were identified in one of five family registries in the US, Canada, England and Australia.

## METHODS

### Subjects

We used five registry sources to ascertain female carriers with germline mutations of BRCA1 or BRCA2, and classified them as cases (those diagnosed with primary invasive cancer of the ovarian epithelium, confirmed by pathology report or death certificate) or controls. The first source (eight cases and 12 controls) was families enrolled with the United Kingdom Consortium for Clinical Cancer Research (UKCCCR) Ovarian Cancer Register. Eligible families were those containing at least two first- or second-degree relatives confirmed with invasive epithelial ovarian cancer. The second source (27 cases and 51 controls) was Australian families registered with the Kathleen Cuningham Foundation Consortium for Research into Familial Breast Cancer (kConFab) (Osborne *et al*, 2000; http://www.kconfab.org). Eligible families contained two living women with breast or ovarian cancer, or one living affected plus one female carrier. The third source (54 cases and 24 controls) was families enrolled in the Gilda Radner Familial Ovarian Cancer Registry at the Roswell Park Cancer Institute in Buffalo, NY, USA. Eligible families contained two or more relatives with a confirmed diagnosis of cancer of the ovary, peritoneum or fallopian tubes. The fourth source (nine cases and 24 controls) was families identified by the Risk Assessment Program at the Fox Chase Cancer Center in Philadelphia, PA, USA. The fifth source (49 cases and 193 controls) was families from the US, Canada and Australia enrolled in the Breast Cancer Family Registry (Breast CFR) (John *et al*, 2004).

We included in the analysis women who were found by genetic testing to carry a germline mutation in BRCA1 or BRCA2 that was predicted to adversely affect protein function (National Human Genome Research Project, 2002) who provided information concerning their reproductive characteristics and oral contraceptive use in a structured questionnaire, and who had not participated as a study subject in any other study of oral contraceptive use and ovarian cancer risk.

### Mutation analysis

In the UKCCCR Ovarian Cancer Register and the GRFOCR Registry, one affected member from each family (hereafter called the index case) was tested for germline mutations in BRCA1 and BRCA2 by a combination of the protein truncation test (PTT) and single-strand conformation analysis/heteroduplex analysis (SSCP/HA), as described previously (Gayther *et al*, 1999). Direct sequence analysis was used to characterise the nucleotide alteration associated with PTT or SSCP/HA variants. When a mutation was identified in an index case, other family members were tested for that mutation by direct sequencing. Women registered with the Fox Chase Cancer Center were tested for mutations using the enzymatic mutation detection assay (Del Tito *et al*, 1998). Mutation testing for members of families enrolled in kConFab (Scott *et al*, 2003) and the Breast CFR (John *et al*, 2004) was undertaken in several different diagnostic laboratories using different strategies including HA, PTT, chemical cleavage of mismatch, allele-specific oligonucleotide hybridisation and direct sequencing.

### Study protocol

Participants provided data on their histories of childbearing, oophorectomy and use of oral contraceptives. The women reported the ages when they started and stopped taking oral contraceptives and their total durations of use. No information from surrogate interviews was included in the analysis.

### Statistical analysis

To each carrier we assigned a reference age, defined as her age at diagnosis if she was a case, or as her age at the earlier of oophorectomy or interview if she was a control. We matched each case to one or more controls. Controls were matched to the case on year of birth (within 3 years), country of residence (Australia, Canada, UK, US) and gene (BRCA1, BRCA2). In addition, the reference ages of controls must have exceeded those of their matched case. Within each matched set, we considered childbirth and oral contraceptive use only prior to the case's reference age. We performed a matched case–control analysis using conditional logistic regression (Breslow and Day, 1980), with parity (0, 1 or 2, 3+ live births) included in the regression models. Since we are testing the null hypothesis of no association between ovarian cancer risk and oral contraceptive use against the alternative hypothesis of reduced risk associated with oral contraceptive use, we report one-tailed *P*-values.

## RESULTS

We were able to find at least one matched control for 114 cases with BRCA1 mutations (the mean number of controls per case was 2.0, with range 1–3) and for 33 cases with BRCA2 mutations (mean number of controls per case was 2.4, with range 1–3). Table 1

**Table 1:** Distribution of matched ovarian cancer case–control sets^a^, according to case's age at diagnosis, year of birth and country of residence, by gene

	**BRCA1**	**BRCA2**	**Total**
	***N* (%)**	***N* (%)**	***N* (%)**
*Case's age at dx* (%)			
<40	18 (16)	3 (9)	21 (14)
40–49	55 (48)	12 (36)	67 (46)
50–59	29 (25)	10 (31)	39 (27)
60+	12 (11)	8 (24)	20 (13)
			
*Case's year of birth* (%)			
<1930	12 (11)	6 (18)	18 (12)
1930–1944	44 (39)	15 (46)	59 (40)
1945+	58 (50)	12 (36)	70 (48)
			
*Case's country of residence*			
Australia	29 (25)	4 (12)	33 (23)
Canada	2 (2)	1 (3)	3 (2)
UK	8 (7)	0 (0)	8 (5)
US	75 (66)	28 (85)	103 (70)
			
Total no. of matched sets	114	33	147

a Controls had intact ovaries at the age when the case was diagnosed, and were matched to cases on gene (BRCA1 or BRCA2), year of birth (±3 years) and country of residence (Australia, Canada, UK, USA).

shows the distributions of matched case–control sets according to age at diagnosis, year of birth and country of residence of the cases, by gene. Most cases were diagnosed between the ages of 40 and 60 years, and most were born after 1930. Cases (and therefore controls) were more likely to carry mutations of BRCA1 than BRCA2 by a factor of more than three.

Table 2

**Table 2:** Characteristics of ovarian cancer cases and control women, by gene

	**BRCA1**		**BRCA2**		**Total**	
	**Cases**	**Controls**	**Cases**	**Controls**	**Cases**	**Controls**
No.	114	225	33	79	147	304
Mean no. live births	2.5	2.5	2.6	2.5	2.5	2.5
Mean age of (years) first birth among parous	23.5	24.6	24	24.5	23.6	24.6
Ever used oral contraceptives (%)	53	63	54	58	53	62
Mean years of oral contraceptive use among users	3.0	4.7	3.1	4.3	3.0	4.6
Prophylactic bilateral oophorectomy (%)	—	15	—	6	—	12
Carrier of Ashkenazi founder mutation^a^ (%)	32	39	27	29	31	36

a Mutations 185 del AG, 5382 ins C or 6174 del T.

shows the distributions of participants according to reproductive characteristics and oral contraceptive use, by gene and case–control status. Compared to controls, cases were less likely to use oral contraceptives and had used them for fewer years. Overall, 31% of ovarian cancer patients and 36% of control women carried one of the two common founder mutations of BRCA1 (185 del AG or 5382 ins C) or the single common founder mutation of BRCA2 (6174 del T).

Table 3

**Table 3:** Ovarian cancer risk according to oral contraceptive use among carriers of BRCA1 or BRCA2 mutations

	**Cases (*N*=147) No. (%)**	**Controls (*N*=304) No. (%)**	**OR^a^**	**CI^b^**
*Any use*^c^				
No	69 (46.9)	116 (38.2)	1.0	—
Yes	78 (53.1)	188 (61.8)	0.85	0.53–1.4
				
*Years of use*				
<1	69 (46.9)	116 (38.2)	1.0	—
1–2	31 (21.1)	40 (13.2)	1.5	0.82–2.9
3–5	14 (9.5)	44 (14.4)	0.69	0.33–1.4
6+	33 (22.5)	104 (34.2)	0.62	0.35–1.1
				
Trend per year of use among users			0.95	0.91–0.99

a OR=odds ratio, adjusted for study centre, parity and age.

b CI=95% confidence interval.

c For at least 1 year.

shows ovarian cancer odds-ratios in relation to oral contraceptive use. The odds-ratio associated with use for at least 1 year, compared to use for less than 1 year or nonuse, was 0.85 (95% confidence interval 0.53–1.4). Also shown are odds-ratios in relation to duration of oral contraceptive use. The odds-ratio among carriers who had used oral contraceptives for 6 or more years compared to those who had used them for less than 1 year was 0.62 (95% confidence interval 0.35–1.1). The data show a trend of decreasing risk with increasing duration of use, with an overall 5% (95% confidence interval 1–9%) reduction in risk per year of use (one-tailed *P*=0.01).

When restricted to BRCA1 mutation carriers, estimates of associations with oral contraceptives use were similar to those shown in Table 3. Estimates based on BRCA2 mutation carriers alone were imprecise, and were not statistically different from those for BRCA1 mutation carriers.

## DISCUSSION

Among women who carry mutations of BRCA1 or BRCA2, we found reduced risk associated with use of oral contraceptives and evidence for increasing risk reduction with increasing duration of use. The reduction in risk of 14% among ever users and 38% among long-term users are consistent with, but somewhat weaker than, reductions observed in the general population. In a pooled analysis of six population-based case–control studies of oral contraceptives and the risk of ovarian cancer in the US, the risk reduction associated with ever use was 34%, and that associated with 6 or more years of use was 70% (Whittemore *et al*, 1992).

Other studies have produced conflicting results on the relationship between ovarian cancer risk and oral contraceptive use among carriers of BRCA1 or BRCA2 mutations. Narod *et al* (1998, 2001a) found risk reductions of magnitude similar to those seen here. The risk reductions did not vary when the authors separated carriers by type of mutation (BRCA1 *vs* BRCA2). In a population-based case–control study of ovarian cancer among women in Northern California, McGuire *et al* (2004) also found an inverse relationship between oral contraceptive use and ovarian cancer risk when comparing patients carrying a BRCA1 mutation with control women from the general population. In contrast, in a similar population-based case–control study of ovarian cancer among women in Israel, Modan *et al* (2001) found no differences in oral contraceptive use when comparing patients carrying one of the three common BRCA1 or BRCA2 founder mutations with control women from the general Israeli population. The discrepancies between these results, if not due to chance or bias, could reflect differences in the populations at risk, the specific types of mutations studied, or study design (Modan and Wacholder, 2001; Narod *et al*, 2001b).

The present results, based on data that have not been reported in any previous studies of oral contraceptive use and ovarian cancer, support previous findings of Narod *et al* (1998, 2001a). Our study and those of Narod *et al* share some limitations. First, they included data only from living affected carriers because of the difficulty of obtaining accurate histories from relatives of deceased patients. These living patients were prevalent cases who reported their prediagnostic oral contraceptive histories some time after their cancer diagnoses. If oral contraceptive use is associated with an altered mortality in women with ovarian cancer, then this selection strategy may result in a biased odds-ratio estimate. Second, most study subjects were members of families with multiple cases of breast and ovarian cancer, and therefore the mutations in the women we have studied may have been those that cause a higher risk of these cancers. Odds-ratios for oral contraceptive uses in these carriers may not pertain to the general population of carriers. Third, some of the unaffected carriers were relatives of ovarian cancer patients. While this design limits potential confounding by ethnicity, a disadvantage is that correlation among sisters in oral contraceptive use could distort both the magnitude of the association and the lengths of confidence intervals. Yet we found only modest correlation between sisters in duration of oral contraceptive use. In addition, our inclusion of control carriers who had undergone prophylactic oophorectomy could overestimate the protection afforded by oral contraceptive use, if these carriers were more likely to use oral contraceptives than other carriers. However, we found no difference in the prevalence of oral contraceptive use between carriers with and without prophylactic oophorectomy.

The studies by Modan *et al* (2001) and McGuire *et al* (2004) avoided these limitations by including only incident cases of ovarian cancer in mutation carriers and unrelated controls. Prevalence of oral contraceptive use among carrier cases was compared to that in control women from the general population. Inferences based on such a comparison are valid only if oral contraceptive use is similar among carriers and noncarriers in the general population. The null findings of Modan *et al* (2001) may have occurred because the oral contraceptive use of carrier cases was compared to that of controls who were older than they were. This difference occurred because controls were age-matched to all cases, whereas the carrier cases were younger than the noncarrier cases, and the analytic comparisons were stratified only in broad 10-year age categories. Thus, the comparison controls were born earlier than the carrier cases, and had less opportunity for long-term exposure to oral contraceptives, which did not become widespread until after 1960. This problem was mitigated in the report of McGuire *et al* (2004) by the more recent birth years of all subjects, and by finer age stratification in the analysis.

In conclusion, we found reduced ovarian cancer risk associated with long-term oral contraceptive use among carriers of BRCA1 or BRCA2 mutations. Both the reduced incidence among ever-users and the trend of decreasing risk with increasing duration of use are consistent with a protective effect for oral contraceptives in these women. If such protection exists, the benefits of oral contraceptive use must be weighed against its possible adverse effects on breast cancer risk. Although there are data to suggest that oral contraceptive use is associated with a small increased breast cancer risk in carriers of BRCA1 (but not BRCA2) mutations (Narod *et al* 2002), this effect was being driven by use prior to the mid 1970s. A more recent population-based study has found no evidence for an increased risk in mutation carriers associated with use of current formulations of oral contraceptives, and evidence that current formulations may even be protective for BRCA1 mutation carriers (Milne *et al*, 2004). Therefore, oral contraceptive use in women with BRCA1 mutations should be considered in light of any planned prophylactic surgery. The current data suggest that women with intact ovaries who undergo prophylactic mastectomy would be good candidates for oral contraceptives. Further data are needed to advise those who undergo prophylactic oophorectomy but not mastectomy.

## References

[bib1] Antoniou A, Pharoah PDP, Narod S, Risch HA, Eyfjord JE, Hopper JL, Loman N, Olsson H, Johannsson O, Borg A, Pasini B, Radice P, Manoukian S, Eccles D, Tang N, Olah E, Anton-Culver H, Warner E, Lubinski J, Gronwald J, Gorski B, Tulinius H, Eerola H, Nevanlinna H, Syrjakoski K, Kalliomeni O-P, Thompson D, Evans C, Peto J, Lalloo F, Evans DG, Easton DF (2003) Average risks of breast and ovarian cancer associated with mutations in BRCA1 and BRCA2 detected in case series unselected for family history: a combined analysis of 22 studies. Am J Hum Genet 72: 1117–1130 12677558 10.1086/375033 PMC1180265

[bib2] Breslow NE, Day NE (1980) Statistical methods in cancer research. Volume I – The analysis of case–control studies. IARC Sci Publ, (32): 5–338 7216345

[bib4] Del Tito Jr BJ, Poff III HE, Novotny MA, Cartledge DM, Walker II RI, Earl CD (1998) Automated fluorescent analysis procedure for enzymatic mutation detection. Clin Chem 44: 731–739 9554483

[bib5] Ford D, Easton DF, Stratton M, Narod S, Goldgar D, Devilee P, Bishop DT, Weber B, Lenoir G, Chang-Claude J, Sobol H, Teare MD, Struewing J, Arason A, Scherneck S, Peto J, Rebbeck TR, Tonin P, Neuhausen S, Barkardottir R, Eyfjord J, Lynch H, Ponder BAJ, Gayther SA, Birch JM, Lindblom A, Stoppa-Lyonnet D, Bignon Y, Borg A, Hamann U, Haites N, Scott RJ, Maugard CM, Vasen H, Seitz S, Cannon-Albright LA, Schofield A, Zelada-Hedman M, Breast Cancer Linkage Consortium (1998) Genetic heterogeneity and penetrance analysis of the BRCA1 and BRCA2 genes in breast cancer families. The Breast Cancer Linkage Consortium. Am J Hum Genet 62: 676–689 9497246 10.1086/301749 PMC1376944

[bib6] Gayther SA, Russell P, Harrington P, Antoniou A, Easton DF, Ponder BAJ (1999) The contribution of germline BRCA1 and BRCA2 mutations to familial ovarian cancer: no evidence for other ovarian cancer-susceptibility genes. Am J Hum Genet 65: 1021–1029 10486320 10.1086/302583 PMC1288234

[bib7] John EM, Hopper JL, Beck JC, Knight JA, Neuhausen SL, Senie RT, Ziogas A, Andrulis IL, Anton-Culver H, Boyd N, Buys SS, Daly MB, Santella RM, Southey MC, Venne VL, Venter DJ, West DW, Whittemore AS, Seminara D, The Breast Cancer Family Registry (2004) The Breast Cancer Family Registry (Breast CFR): an infrastructure for cooperative multinational, interdisciplinary and translational studies of the genetic epidemiology of breast cancer. Breast Cancer Res 6: R375–R389, (http://www.cfr.epi.uci.edu) 15217505 10.1186/bcr801 PMC468645

[bib8] La Vecchia C, Franceschi S (1999) Oral contraceptives and ovarian cancer. Eur J Cancer Prev 8: 297–304 10493304 10.1097/00008469-199908000-00005

[bib21] McGuire V, Felberg A, Mills M, Ostrow KL, DiCioccio RA, John EM, West DW, Whittemore AS (2004) Relation of contraceptive and reproductive history to ovarian cancer risk in carriers and noncarriers of BRCA1 gene mutations. Am J Epidemiol 160: 613–618 15383404 10.1093/aje/kwh284

[bib9] Milne RL, Knight JA, John EM, Dite GS, Balbuena R, Ziogas A, Andrulis IL, West DW, Southey MC, Giles GG, McCredie MRE, Hopper JL, Whittemore AS, for the Breast Cancer Family Registry (2004) Oral contraceptive use and risk of early-onset breast cancer in carriers and non-carriers of BRCA1 and BRCA2 mutations. Cancer Epidemiol Biomarkers Prev (in press) 10.1158/1055-9965.EPI-04-0376 15734957

[bib11] Modan B, Hartge P, Hirsh-Yechezkel G, Chetrit A, Lubin F, Beller U, Ben-Baruch G, Fishman A, Menczer J, Ebbers SM, Tucker MA, Wacholder S, Struewing JP, Friedman E, Piura B (2001) Parity, oral contraceptives, and the risk of ovarian cancer among carriers and noncarriers of a BRCA1 or BRCA2 mutation. N Engl J Med 345: 235–240 11474660 10.1056/NEJM200107263450401

[bib10] Modan B, Wacholder S (2001) Parity, oral contraceptives, and the risk of ovarian cancer among carriers and noncarriers of a BRCA1 or BRCA2 mutation. N Engl J Med 345: 1707 (letter) 10.1056/NEJM200107263450401 11474660

[bib12] Narod SA, Dube MP, Klijn J, Lubinski J, Lynch HT, Ghadirian P, Provencher D, Heimdal K, Moller P, Robson M, Offit K, Isaacs C, Weber B, Friedman E, Gershoni-Baruch R, Rennert G, Pasini B, Wagner T, Daly M, Garber JE, Neuhausen SL, Ainsworth P, Olsson H, Evans G, Osborne M, Couch F, Foulkes WD, Warner E, Kim-Sing C, Olopade O, Tung N, Saal HM, Weitzel J, Merajver S, Gauthier-Villars M, Jernstrom H, Sun P, Brunet JS (2002) Oral contraceptives and the risk of breast cancer in BRCA1 and BRCA2 mutation carriers. J Natl Cancer Inst 94: 1773–1779 12464649 10.1093/jnci/94.23.1773

[bib13] Narod SA, Risch H, Moslehi R, Dorum A, Neuhausen S, Olsson H, Provencher D, Radice P, Evans G, Bishop S, Brunet JS, Ponder BA (1998) Oral contraceptives and the risk of hereditary ovarian cancer. N Engl J Med 339: 424–428 9700175 10.1056/NEJM199808133390702

[bib14] Narod SA, Sun P, Ghadirian P, Lynch H, Isaacs C, Garber J, Weber B, Karlan B, Fishman D, Rosen B, Tung N, Neuhausen SL (2001a) Tubal ligation and risk of ovarian cancer in carriers of BRCA1 or BRCA2 mutations: a case–control study. Lancet 357: 1467–1470 11377596 10.1016/s0140-6736(00)04642-0

[bib15] Narod SA, Sun P, Risch HA (2001b) Ovarian cancer, oral contraceptives, and BRCA mutations. N Engl J Med 345: 1706–1707 11759652 10.1056/NEJM200112063452312

[bib22] National Human Genome Research Project (2002) Breast cancer information core: an open access on-line breast cancer mutation data base, 2002 (Accessed 15 May, 2004, at http://research.nhgri.nih.gov/bic/)

[bib16] Osborne RH, Hopper JL, Kirk JA, Chenevix-Trench G, Thorne HJ, Sambrook JF, kConFab: a research resource of Australasian breast cancer families (2000) Kathleen Cuningham Foundation Consortium for Research into Familial Breast Cancer. Med J Aust 172: 463–464 10870547 10.5694/j.1326-5377.2000.tb124064.x

[bib17] Scott CL, Jenkins MA, Southey MC, Davis TA, Leary JA, Phillipes K-A, Hopper JL, for the Kathleen Cuningham Foundation for Familial Breast Cancer (kConFab) (2003) Average age-specific risk of breast cancer associated with germline mutations in BRCA1 and BRCA2 estimated from families attending Australian Family Cancer Clinics. Hum Genet 112: 542–551 12601471 10.1007/s00439-003-0908-6

[bib18] Struewing JP, Hartge P, Wacholder S, Baker SM, Berlin M, McAdams M (1997) The risk of cancer associated with specific mutations of BRCA1 and BRCA2 among Ashkenazi Jews. N Engl J Med 336: 1401–1408 9145676 10.1056/NEJM199705153362001

[bib19] Ursin G, Henderson BE, Haile RW, Pike MC, Zhou N, Diep A, Bernstein L (1997) Does oral contraceptive use increase the risk of breast cancer in women with BRCA1/BRCA2 mutations more than in other women? Cancer Res 57: 3678–3681 9288771

[bib20] Whittemore AS, Harris R, Itnyre J, Collaborative Ovarian Cancer Group (1992) Characteristics relating to ovarian cancer risk: collaborative analysis of twelve US case–control studies. II. Invasive epithelial ovarian cancers in white women. Am J Epidemiol 136: 1184–1203 1476141 10.1093/oxfordjournals.aje.a116427

